# Fabrication and characterization of high-sensitivity, wide-range, and flexible MEMS thermal flow velocity sensors

**DOI:** 10.1038/s41378-024-00740-2

**Published:** 2024-07-22

**Authors:** Min Li, Guangzhao Qin, Chen Jia, Danyu Zhang, Zhikang Li, Xiangguang Han, Shusheng Xu, Libo Zhao, Guoxi Luo, Cunlang Liu, Ping Yang, Qijing Lin

**Affiliations:** 1 State Key Laboratory for Manufacturing Systems Engineering, International Joint Laboratory for Micro/Nano Manufacturing and Measurement Technologies, Xi’an Jiaotong University (Yantai) Research Institute for Intelligent Sensing Technology and System, Xi’an Jiaotong University, Xi’an, 710049 China; 2 School of Instrument Science and Technology, Xi’an Jiaotong University, Xi’an, 710049 China; 3 Shandong Laboratory of Advanced Materials and Green Manufacturing at Yantai, Yantai, 264000 China; 4 Brightstone Innovation (Yantai) Research Institute for Micronano Sensing Technology, Yantai, 264006 China; 5 Xi’an Jingwei Sensing Technology Co., Ltd., Xi’an, 712000 China

**Keywords:** Electrical and electronic engineering, Sensors

## Abstract

With the rapid development of various fields, including aerospace, industrial measurement and control, and medical monitoring, the need to quantify flow velocity measurements is increasing. It is difficult for traditional flow velocity sensors to fulfill accuracy requirements for velocity measurements due to their small ranges, susceptibility to environmental impacts, and instability. Herein, to optimize sensor performance, a flexible microelectromechanical system (MEMS) thermal flow sensor is proposed that combines the working principles of thermal loss and thermal temperature difference and utilizes a flexible cavity substrate made of a low-thermal-conductivity polyimide/SiO_2_ (PI/SiO_2_) composite porous film to broaden the measurement range and improve the sensitivity. The measurement results show that the maximum measurable flow velocity can reach 30 m/s with a resolution of 5.4 mm/s. The average sensitivities of the sensor are 59.49 mV/(m s^−1^) in the medium-to-low wind velocity range of 0–2 m/s and 467.31 mV/(m s^−1^) in the wind velocity range of 2–30 m/s. The sensor proposed in this work can enable new applications of flexible flow sensors and wearable devices.

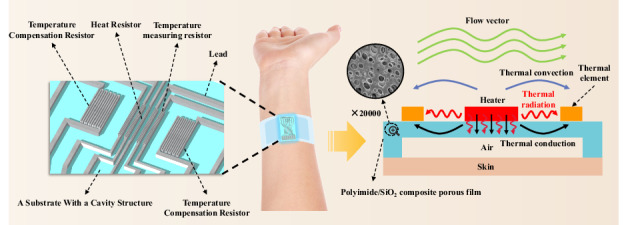

## Introduction

The flow velocity is an essential physical quantity in various industrial and daily applications^1^. In the past few decades, with the development of the aerospace, military, and biomedical fields, increased requirements have been promoted for flow velocity sensors^2–4^. For example, the requirements for measurement accuracy are becoming increasingly high, the measurement environment is becoming increasingly harsh, and the measurement range is increasing^5,6^. Traditional flow velocity sensors can no longer meet these requirements. Thermal flow velocity sensors have attracted considerable attention due to their simple structures, high accuracies, and small sizes^7,8^. With the rapid development of the Internet of Things, electronic skin, and microelectromechanical system (MEMS) technology, MEMS thermal flow velocity sensors have gradually become mainstream owing to their unique advantages of small sizes, high integration capabilities, and simple measurement methods^9–12^.

Efforts have been made to further develop and improve MEMS thermal flow velocity sensors. For example, Cubukcu et al.^13^ designed a thermal flow velocity sensor; the sensitivity of amorphous germanium as a sensitive resistor was 4.12 Ks·m^−1^, and the resolution of the sensor was 0.1 mm/s. Balakrishnan et al.^14^ prepared a highly sensitive thermal flow velocity sensor using a 3C-SiC sensitive material with a range of 0.2–9.0 m/s. Shen et al.^15^ reported a thermal flow velocity sensor for leeward measurements in the range of 0–8 m/s with an accuracy of 0.3 m/s. Cho et al.^16^ developed a flexible microthermal flow sensor on a flexible substrate with a flow rate ranging from 25–200 mL/min. However, the detection principle of these devices was based on a single device, limiting the measurement range of velocity. In addition, polyimide (PI) is widely used in the construction of thermal flow velocity sensors due to its excellent properties, such as its high mechanical strength, low-thermal conductivity, and good temperature resistance^17–20^. To further reduce the thermal conductivity and improve the temperature resistance, research on modifying polyimides with organic silicon has gradually become a popular topic in recent years. To date, researchers^21–24^ have focused mainly on adding organosilicon polymers to PI solutions through simple mechanical stirring or ultrasonication; these materials exhibit poor uniformity and are prone to particle aggregation, resulting in limited performance improvement. Introducing organic silicon onto the main chain of PI through chemical modification can effectively improve compatibility and uniformity. Therefore, their potential application in flow velocity sensors should be investigated to explore the possibility of improving device performance.

Herein, a new MEMS thermal flow velocity sensor based on a porous polyimide/SiO_2_ (PI/SiO_2_) composite substrate was manufactured by combining the dual detection principles of thermal temperature difference and thermal loss, thus effectively expanding the measurement flow velocity range and improving the sensitivity of the device by designing a cavity structure and reducing the substrate thermal conductivity. The optimized and flexible MEMS thermal flow velocity sensor displayed a maximum measurement flow velocity of 30 m/s with a resolution of 5.4 mm/s and an average sensitivity of 467.31 mV/(m s^−1^), which was 39% greater than that of the sensor with a pure PI substrate. All the findings above indicated that the developed device had good practical application prospects for constructing a velocity measurement system.

## Design of the thermal flow velocity sensor

**Fig. 1:**
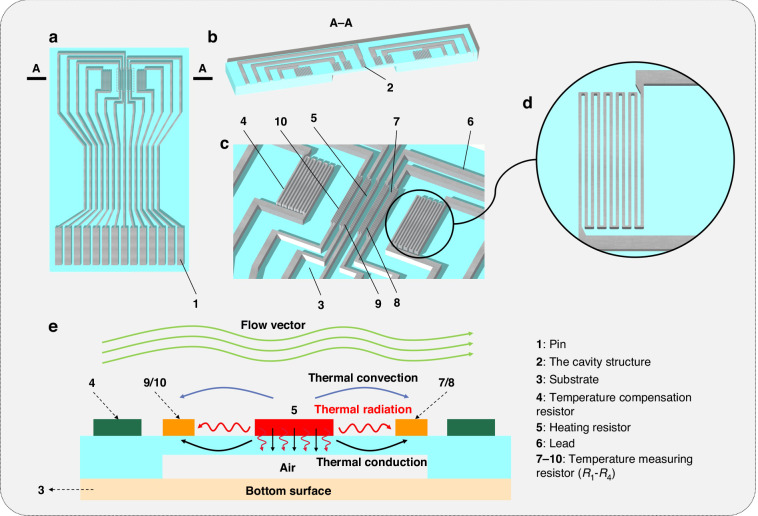
Design of the sensor. **a** Schematic diagram of the overall structure. **b** Section view. **c** Detailed diagram of the key sensing structure. **d** Enlarged view of a single resistor component in a sensor. **e** Diagram of the working principle

A schematic diagram of the proposed thermal flow velocity sensor is shown in Fig. 1. The sensor is composed of a PI/SiO_2_ substrate with a cavity structure, a Pt heating resistor, two pairs of Pt temperature measurement resistors, Pt temperature compensation resistors, lead wires, and bonding pads (Fig. 1a). The overall thickness of the device is 7 μm, and the cavity height is 5 μm, which can be observed from a cross-sectional view of the sensor cut horizontally from the middle of the cavity (Fig. 1b). The heating resistor is designed as an 800 × 40 μm serpentine structure with a wire width of 8 μm, providing a uniform and constant temperature field. Two pairs of temperature measurement resistors are designed at distances of 300 and 500 μm from the heating resistor. The resistor pairs (*R*_*2*_ and *R*_*3*_) are closest to the central heating resistor, and the resistor pairs (*R*_*1*_ and *R*_*4*_) are on their sides (Fig. 1c). Additionally, a pair of temperature compensation resistors is designed to measure the fluid temperature for temperature compensation (Fig. 1d). The dimensions of the temperature measurement resistors are 800 × 72 μm, and those of the temperature compensation resistors are 800 × 105 μm. Both resistors have wire widths of 8 μm and exhibit serpentine structures. As shown in Fig. S1, the temperature coefficient of resistance (TCR) of the Pt heating resistor is 0.1583%/°C, and that of the Pt temperature-measuring resistor is 0.1609%/°C. The pin position of the device is designed according to the standard flip type FPC connector, with a pad spacing of 1 mm, a total width of 15 mm, and a total thickness of 0.3 mm. This design facilitates connection and standardized docking with the 14PIN FPC to solve the connection problem of flexible circuits in the later stage.

The detection principle of this sensor is shown in Fig. 1e. This principle combines the concepts of thermal loss and thermal temperature difference, effectively expanding the measurement range. The thermal loss principle is based on the heat dissipation effect, in which the heating resistor is directly placed in a flowing fluid. Because the heating resistance of the sensor is compensated through a circuit to ensure a constant temperature difference between the heating resistance and the fluid, if the flow velocity changes, the heating resistance must be changed to ensure a constant temperature difference between the two. Therefore, by obtaining the power of the heating resistance, the flow velocity of the fluid can be calculated. The thermal temperature difference principle is based on the thermal distribution effect. When the fluid flows over the surface of the heating resistor, it causes an asymmetric temperature distribution in the fluid. The sensor measures the difference in the resistance values of the two temperature-sensing resistors on each side of the heating resistor to determine the flow velocity.

Due to the limited measurement range of the thermal temperature difference principle, it is only applicable below a certain critical velocity. Beyond this critical velocity, the sensor starts utilizing the thermal loss principle, which offers greater linearity and sensitivity than the thermal temperature difference principle. The thermal loss principle has the problem of low measurement accuracy at low flow velocities; thus, combining the two can effectively expand the measurement range. Based on the simulation results (Fig. S2), by considering the sensitivity and measurable range of low-velocity measurements, the distances between the two pairs of temperature-measuring resistors and the heating resistor are 300 and 500 μm, respectively. Furthermore, when the flow velocity exceeds 2 m/s, the thermal loss principle is utilized, and when the flow velocity is below 2 m/s, the thermal temperature difference principle is applied.

## Experimental procedures

### Preparation of the PI/SiO_2_ composite porous substrate

The main reagents used were *N*,*N*-dimethylacetamide (DMAC): chemically pure, Shanghai

Aladdin Biochemical Technology Co., Ltd.; pyromellitic dianhydride (PMDA): purity ≥99%, Shanghai Aladdin Biochemical Technology Co., Ltd.; 4,4’-diaminodiphenyl ether (ODA): chemically pure, Shanghai Aladdin Biochemical Technology Co., Ltd.; tetraethyl orthosilicate (TEOS): chemically pure, Damao Chemical Reagent Factory; γ-aminopropyl triethoxysilane (APTES): chemically pure, Shanghai Aladdin Biochemical Technology Co., Ltd.; glacial acetic acid (Ac): purity ≥99%, Fuyu Chemical; triethylamine (TEA): chemically pure, Fuyu Chemical; and anhydrous ethanol (EtOH): purity ≥99.7%, Shanghai Aladdin Biochemical Technology Co., Ltd.

**Fig. 2:**
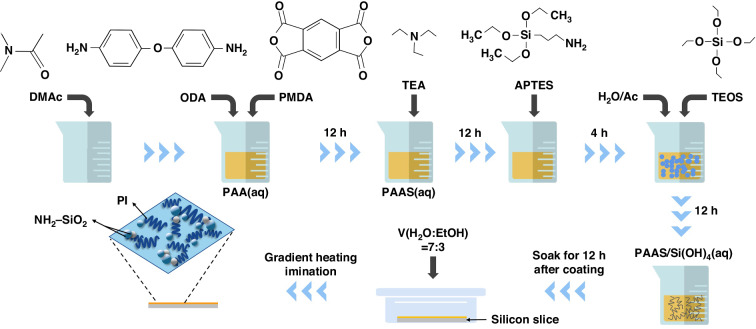
Preparation of the low-thermal-conductivity substrate

As shown in Fig. 2, the materials were prepared as follows. A polyamic acid (PAA) solution was prepared by dissolving ODA in DMAc solvent and adding PMDA in an equimolar ratio. The mixture was stirred for 12 h to obtain a PAA solution with a solid content of 16%. TEA was added to an equal amount of carboxylic acid, and the mixture was stirred for 12 h to obtain a viscous PAAS solution. The crosslinking agent APTES was added dropwise via stirring and crosslinked with the PAAS chain segment. Then, TEOS was added dropwise. After 1 h, water and glacial acetic acid were continuously added to initiate the hydrolysis of TEOS. The ratios of the various reagents are shown in Table 1. The mixture was stirred for 12 h to prepare the PAAS/Si(OH)_4_ composite sol. The sol was applied onto a clean silicon wafer followed by immersion in a gelation solution (V (water): V (anhydrous ethanol) = 7:3) for gelation. After 12 h the silicon slices were removed. After the membrane was completely dried, the PI/SiO_2_ composite porous film was obtained by increasing the temperature and thermal amination.

**Table 1:** Composition of the main reagent

DMAc/g	PMDA/g	ODA/g	APTES/g	TEOS/g	TEA/g	H_2_O/g	Ac/g
47.25	4.3077	4.6923	0.4143	7.8	0.65	2.7	0.75

### Sensor manufacturing process

**Fig. 3:**
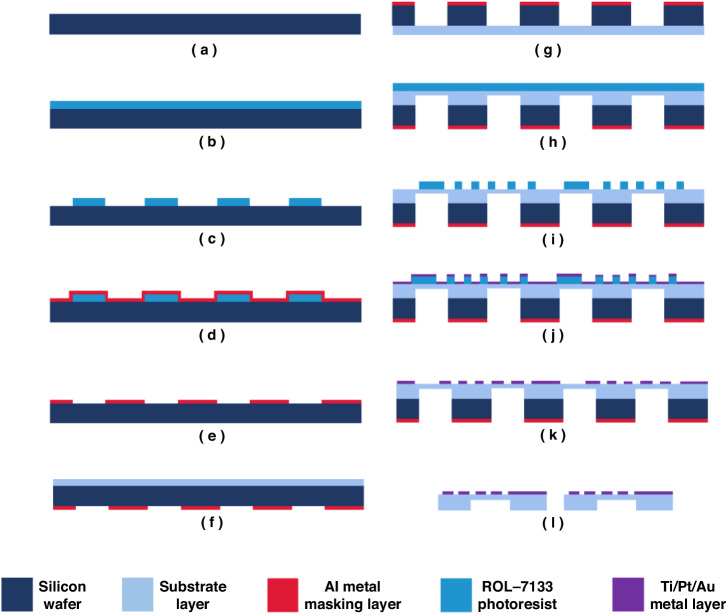
Fabrication of the sensors

The fabrication of the sensor shown in Fig. 3 is as follows. (a–b) A ROL-7133 photoresist was spin-coated on the surface of the silicon wafer. (c–e) An Al sacrificial layer was sputtered as a masking layer for subsequent etching. (f) The PAAS/Si(OH)_4_ composite solution was spin-coated on the other side of the silicon wafer. Then, the gradient temperature method was used to imidize the PI/SiO_2_ composite porous membranes. Herein, a flexible PI/SiO_2_ thin film was chosen as the substrate owing to its low-thermal conductivity and good thermal stability. (g) Deep silicon etching was performed using inductively coupled plasma (ICP) until only the flexible substrate remained at the cavity on the silicon wafer surface. (h) The solution was spun onto the substrate again and subjected to thermal amination. After the second spin coating process, the film thickness significantly increased. Ion beam etching was performed, and the remaining thickness of the flexible substrate was approximately 2 μm. (i–k) A Ti layer with a thickness of approximately 0.05 μm was deposited on the PI/SiO_2_ composite porous film as the adhesive layer using the DC sputtering method, and then a Pt metal layer with a thickness of ~0.2 μm was deposited to prepare the thermal resistance component and corresponding wires. Pt was used for forming heating elements and sensing units due to its high-temperature coefficient of resistance^25^. Then, an Au layer (~0.05-μm thick) was sputtered to improve the electrical connection between the chip and the external circuits. (l) Laser-cutting technology was used to cut the chip and release the flexible sensor (Sensor II). For comparison, Sensor I was prepared using the same process except that the substrate material was replaced with a pure PAA solution, resulting in a pure PI film, and no substrate cavity structure was fabricated.

**Fig. 4:**
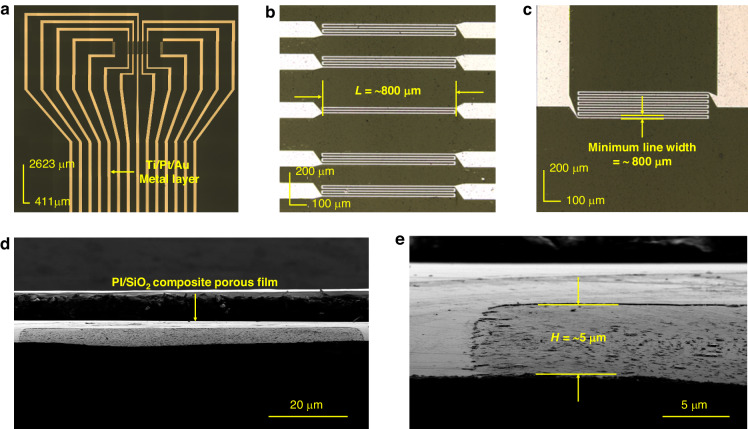
Overall and partial magnified view of the as-fabricated thermal flow sensor. Top views of **a** the upper half of the sensor. Magnified views of the **b** thermal resistor and temperature measurement resistor and **c** temperature compensation resistor. Overall diagram (**d**) and locally enlarged diagram (**e**) of the cavity structure

Laser confocal microscopy (OLS4000) imaging of the as-fabricated sensor (Sensor II) is displayed in Fig. 4a–c; the whole chip had a length of 15 mm and a width of 8 mm. The length of the resistance wire was 800 μm, and the minimum line width reached 8 μm. To observe the cavity in the substrate, the sensor was horizontally cut from the center of the cavity, as shown in the field emission scanning electron microscopy (SU8010) images in Fig. 4d, e. The depth of the cavity at the cross-section was ~5 μm.

### Characterization

**Fig. 5:**
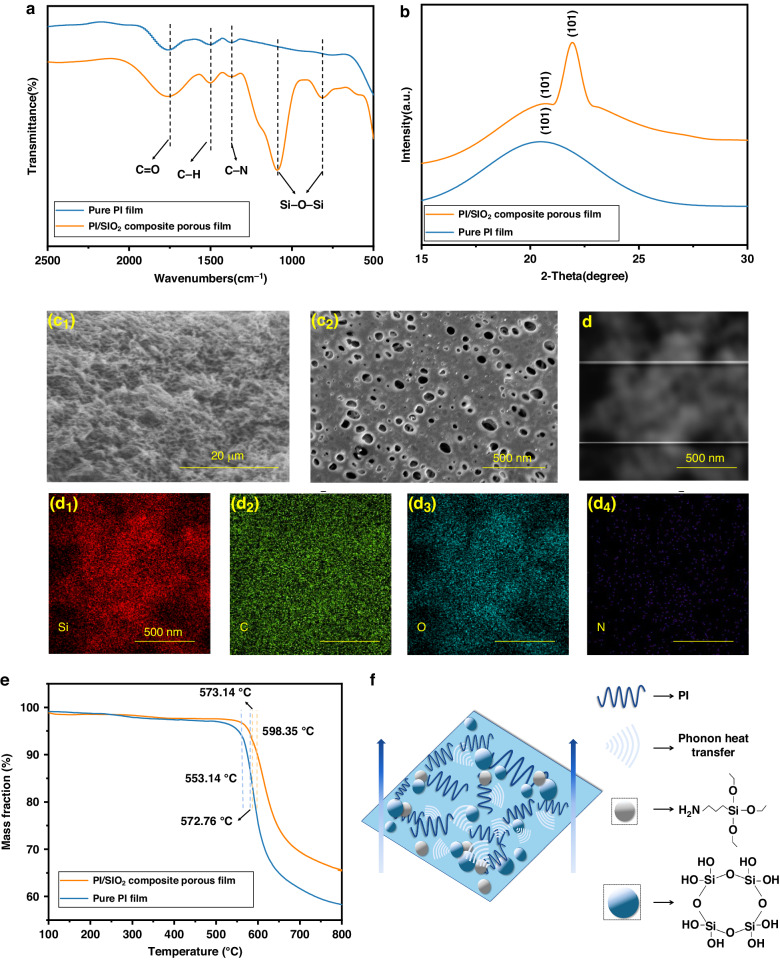
Characterization of the substrate. **a** FTIR spectra of the PI/SiO_2_ composite porous films and pure PI films. **b** XRD patterns of the PI/SiO_2_ composite porous films and pure PI films. **c**_1_ SEM images of the PI/SiO_2_ composite porous films at different magnifications: (magnification 500×); **c**_2_: (magnification 20,000×). Elemental analysis (**d**) and corresponding EDS analysis of Si (**d**_1_), C (**d**_2_), O (**d**_3_), and N (**d**_4_). **e** TG curves of the PI/SiO_2_ composite porous films and pure PI films. **f** Phonon heat transfer mechanism

To confirm the presence of amorphous silica in the polyimide matrix, infrared analysis was performed on the samples. In the infrared spectrum of the PI/SiO_2_ composite porous film displayed in Fig. 5a, 1776 cm^−1^ is the C=O vibration absorption peak, 1500 cm^−1^ is the C-H characteristic absorption peak, and 1373 cm^−1^ is the C-N bond absorption peak. According to the figure, the polyamide acid characteristic absorption peaks disappeared at 1660 and 1550 cm^−1^, respectively, indicating that the composite porous film had basically solidified. The characteristic peaks of Si-O-Si was observed at approximately 1080 and 820 cm^−1^. Additionally, to gain insight into the chemical composition, the composite porous film was ground into powder for XRD analysis. As shown in Fig. 5b, both samples exhibited a carbon peak at 20.8°, while the powder composed of PI/SiO_2_ composite porous film exhibited a significant peak at 21.8°; this peak indicated the presence of amorphous silica within the polyimide matrix^26^.

Moreover, the morphology of the substrate structure was analyzed. Figure 5c_1_, c_2_ show the lack of an obvious silicon ball structure inside the PI/SiO_2_ composite porous film, which instead formed a honeycomb-like porous layered structure. This finding indicates that most SiO_2_ was successfully connected to the PI matrix skeleton through chemical crosslinking, and the compatibility between SiO_2_ and the PI matrix was good, resulting in favorable bonding characteristics. Figure 5d shows an EDS analysis of the powder sample, presenting the elemental distributions of Si, C, O, and N. Si and O were uniformly dispersed throughout the whole structure. Based on the above results, it was concluded that uniformly dispersed amorphous silica was generated in the polyimide matrix.

Figure 5e shows the TG curves of the PI/SiO_2_ composite porous film and pure PI film. The thermal weight loss rate of the composite porous film began to increase at 552.79 °C, with a weight loss of 5% at 573.14 °C and 10% at 598.35 °C. The weight loss of the pure PI film accelerated at 539.95 °C and decreased by 5 and 10% at 553.14 and 572.76 °C, respectively. Evidently, the thermal stability of the PI/SiO_2_ composite porous film was significantly better than that of the pure PI film. The enhanced thermal stability of the PI/SiO_2_ composite porous film was attributed to the homogeneous dispersion of amorphous SiO_2_ in PI^27,28^, which formed an increasingly uniform and compact thermal resistance network. Additionally, the C-H bonds formed between amorphous SiO_2_ and PI could absorb some heat upon breakage, thereby improving thermal stability^29^.

Additionally, thermal conductivity testing of the PI/SiO_2_ composite porous film was carried out. The results indicated that under three repeated tests, the thermal conductivity of the PI/SiO_2_ composite porous film was ~0.077 W/(m·K). In contrast, according to a previous study^30^, the thermal conductivity of the pure PI film was 0.371 W/(m·K), revealing that the thermal conductivity of the PI/SiO_2_ composite porous film was lower than that of the pure PI film. This difference was attributed to the effects of crosslinking and chain disorder on a molecular chain and lattice vibrations, as illustrated in Fig. 5f, which depicts the phonon heat transfer mechanism. After crosslinking the molecular chains of the polyimide framework with amorphous SiO_2_, the crosslinking density increased, resulting in a decrease in molecular crystallinity. During heat transfer, the molecular chains were constrained by the crosslinking points, leading to a reduction in chain orientation^20–23,31^. This phenomenon further intensifies the scattering of phonons by molecular chain vibrations at the polyimide–SiO_2_ interface, reducing the number of ordered transmission channels for the phonon and increasing the thermal resistance at the interface of the material. As a result, the thermal conductivity of the PI matrix decreased from 0.371 W/(m·K) to 0.077 W/(m·K) in the PI/SiO_2_ composite porous film.

### Testing of the thermal flow velocity sensors

**Fig. 6:**
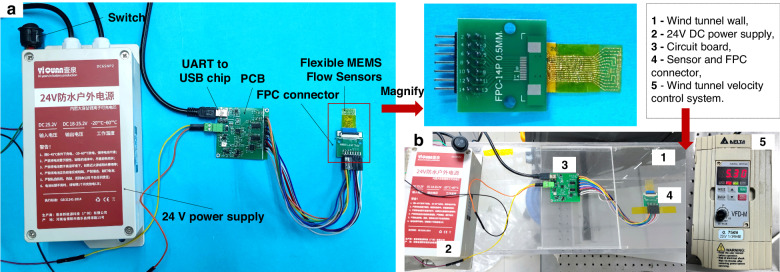
Testing of the flow velocity sensor. **a** Overall assembly of the testing system. **b** Experimental apparatus for conducting wind velocity input measurements

The overall structure of the flow velocity sensor testing system is illustrated in Fig. 6a. This system consists of five components: a flexible sensor, an FPC connector, a 24 V constant voltage source, an FPC circuit board, and upper computer software. The core part of the FPC circuit board was the multichannel digital output circuit system. In the supplementary materials, a detailed explanation of the multichannel digital output circuit system is provided (Figs. S3–S10). The multichannel digital processing unit automatically switched between three sampling signals*—V*_*1*_, *V*_*2*_, and *V*_*3*_—based on the calibrated velocity data, and it seamlessly generated a single output flow velocity signal. Herein, *V*_*1*_ represents the voltage of the high-velocity output system in a measurement range of 2–30 m/s. The signal was generated by the heating resistor. The measurement ranges of the two low-velocity output systems were divided into extremely low velocity (<0.9 m/s) and low velocity (0.9–2 m/s). Signals were generated by the outer temperature measurement resistor pair (*R*_*1*_, *R*_*4*_) and the inner temperature measurement resistor pair (*R*_*2*_, *R*_*3*_), which are represented by *V*_*2*_ and *V*_*3*_, respectively. The sensor was connected to the circuit board via the FPC connector. The 24 V constant voltage source supplied power to the heating resistor of the sensor. The circuit board recorded the voltage difference of the bridge from the ADC chip and transmitted it to the upper computer through the UART-to-USB interface on the circuit board for data transmission and connection with the computer.

The overall assembly of the experimental apparatus for conducting wind velocity input measurements is shown in Fig. 6b. The range and error calibration of the flow velocity sensor were conducted in a self-developed calibration system platform. The calibration system platform comprised an adjustable wind tunnel system (GTI Calibration Wind Tunnel MODEL X5606 series), a digital anemometer (model GM816, used for wind velocity calibration), a flexible flow velocity sensor with its measurement and control circuit, and a multichannel sampling circuit system based on the STM32 development board.

During the wind velocity measurement, the adjustable wind tunnel system generated fluid flow that was directed toward the flexible flow velocity sensor through a pipeline. The sensor samples and the standard flow velocity sensor were placed in parallel positions on the inner wall of the wind tunnel. The flow velocity sensors were connected via wire data loggers, and the wind tunnel system displayed the real-time set wind velocity. The wind velocity gradually increased from low to high by adjusting the knob on the wind tunnel system. The synchronized multichannel sampling circuit system recorded the sensor outputs in real-time.

**Fig. 7:**
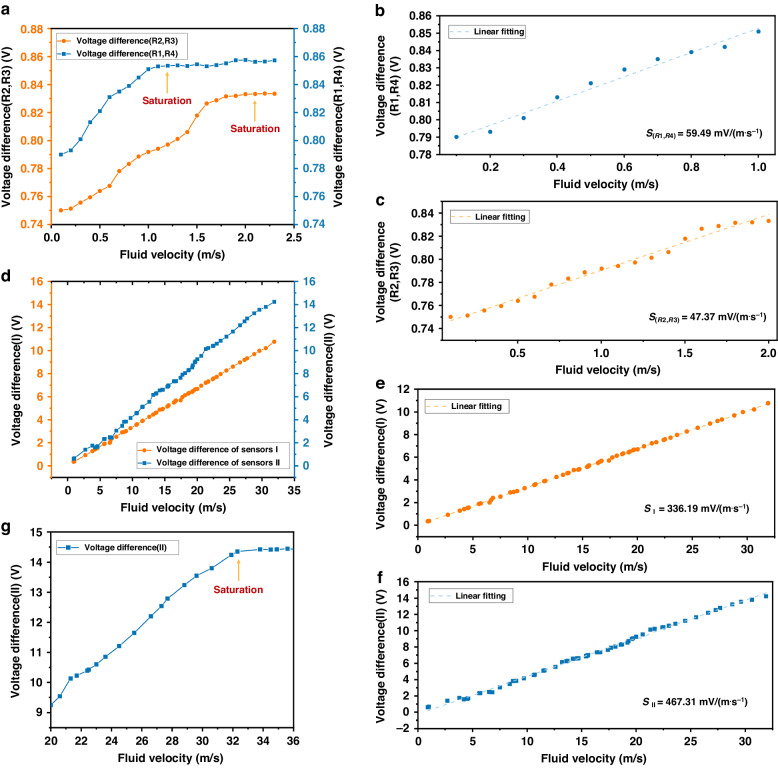
Performance of the sensor. **a** Relationship between the output voltage and the wind velocity. **b**, **c** Sensitivity curve of Sensor II for velocity measurement at low velocity. **d** Relationships between the output voltages of the high-velocity measurement systems of Sensor I and Sensor II and the wind velocity. **e**, **f** Sensitivity curves of the high-velocity outputs of Sensor I and Sensor II. **g** Corresponding wind velocity when the output voltage of the high-speed measurement system of Sensor II reached saturation

The sensitivity of the low-velocity measurement system of Sensor II was tested at an ambient temperature of 25 °C. The wind velocity test range was 0–30 m/s. The relationship between the output voltage of the low-velocity circuit and the wind velocity for Sensor II is shown in Fig. 7a. At low wind velocities, there were two distinct outputs corresponding to the two pairs of temperature measurement resistors that were symmetrically distributed on either side of the heating resistor: *V*_*2*_ and *V*_*3*_. The resistor pairs that were far from the heating resistor exhibited larger temperature differences and higher output voltages than those close to the heating resistor. By calculating the slopes of the output voltage *V*_*2*_ and *V*_*3*_ curves for the two different resistor pairs in Fig. 7a, the sensitivity curve for the low-velocity flow velocity measurement could be obtained, as demonstrated in Fig. 7b, c. When the critical velocity was not reached, the output voltage curves of each pair of temperature-measuring resistors presented good linear relationships. Additionally, there was an obvious disparity in the output voltage range of each of the two pairs of temperature measurement resistors. The analyses of the sensitivity curves in Fig. 7b, c revealed that different temperature measurement resistor pairs exhibited distinct measurement sensitivities within various flow velocity ranges. Further examination yielded the following conclusions. At an extremely low flow velocity range (0–0.9 m/s), the temperature measurement resistor pair (*R*_*1*_, *R*_*4*_) exhibited the highest sensitivity of 54.49 mV/(m·s^−1^). In the range of extremely low to low flow velocities (0.9–2 m/s), the temperature measurement resistor pair (*R*_*2*_, *R*_*3*_) demonstrated the highest sensitivity of 47.37 mV/(m·s^−1^).

Figure 7d depicts the relationship between the output voltage *V*_*1*_ of the high-velocity measurement system of Sensor I and Sensor II and the wind velocity. Figure 7e, f show the sensitivity curves obtained by fitting and calculating the high-velocity output system. From the figures, the following conclusions were drawn. The sensitivity of Sensor II was approximately 467.31 mV/(m s^−1^) in the medium-to-high wind velocity range (2–30 m/s). Additionally, the comparison in the figures revealed that the sensor substrate material, which featured a cavity structure and utilized a PI/SiO_2_ composite porous film, exhibited an average sensitivity that was 39% greater than that of the sensor employing a PI film in the high wind velocity range, beneficial to the relatively lower thermal conductivity. Figure 7g shows that the output voltage of Sensor II reached saturation at a wind velocity of 31.8 m/s. To ensure measurement reliability, the maximum range of speed measurement was ensured to be no less than 30 m/s.

**Fig. 8:**
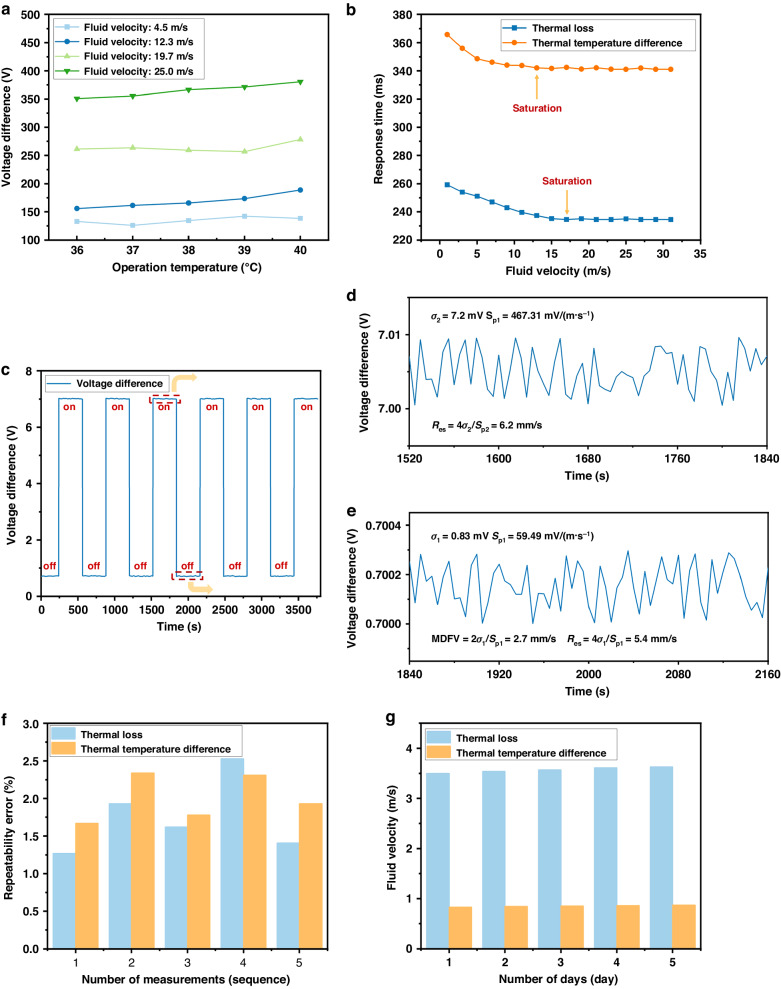
Performance of the sensor. **a** Relationships between the output voltage and temperature at different flow velocities. **b** Response times at different flow velocities. **c**–**e** *R*_*es*_ and MDFV testing. **f** Repeatability error test. **g** Stability test

Sensor II was placed under different ambient temperature conditions to test its ability to suppress temperature drift. The sensors mainly relied on temperature compensation circuits to suppress temperature drift. Specific details about this process are in the supplementary materials. The temperature that a human body could reach ranged from 36 to 40 °C, and this range was selected for testing. Figure 8a shows that the output voltage of the sensor changed as the temperature increased. This phenomenon was caused by the limitation of the thermal sensing mechanism. However, the maximum fluctuation did not exceed 20 mV, and the standard deviation of the sensor output was less than 2%. From these experimental results, it was concluded that the temperature compensation resistor structure could effectively suppress the temperature drift phenomenon.

The dynamic response performance characteristics of the sensors in the application environment were tested and analyzed. Sensor II was placed in the adjustable wind tunnel system, and the real-time output voltage from the wind tunnel was recorded through upper computer software; the wind tunnel wind velocity was continuously increased, and the above process was repeated. The time when the output voltage of the sensor increased to a stable value of 90% was defined as the response time. Due to the differences in the response speeds between the thermal temperature difference and thermal loss principles, the response times of both were recorded simultaneously. Figure 8b shows the average response time test results of the sensor at different flow velocities. When the flow velocity was 2 m/s, the response time of the thermal temperature difference principle was 358 ms. When the flow velocity was 18 m/s, the response time of the thermal loss principle was 235 ms. As the flow velocity increased, the response time continuously decreased and tended to stabilize. The thermal temperature difference principle of the sensor had a longer response time than the thermal loss principle. This disparity arose because when measuring based on the thermal loss principle, the heating resistor responded directly to the action of the fluid. When measuring based on the thermal temperature difference principle, the temperature measurement resistor had to first sense the fluid temperature and then slowly respond.

To address the noise caused by natural and forced convection, long-term reliability testing was conducted on Sensor II, as shown in Fig. 8c. The output of the sensor was recorded under the conditions of a wind tunnel that was switched on (wind velocity of 8.4 m/s) and off (wind velocity of 0 m/s). Through an analysis of time series data, the root mean square (RMS) noise (i.e., standard deviation) measured by the flow velocity sensor under zero-flow conditions was found to be 0.83 mV. The minimum detectable flow velocity (MDFV) and resolution of the flow velocity sensor were determined as follows^32^:

1 $$MDFV=2{V}_{rms}/{S}_{P};{R}_{es}=4{V}_{rms}/{S}_{P}$$

where *V*_rms_ is the root mean square of the voltage and *S*_*p*_ is the sensor sensitivity at this flow velocity.

Therefore, the inherent *MDFV* and flow velocity resolution of Sensor II were 2.7 mm/s and 5.4 mm/s, respectively. In addition, at an input flow of 8.4 m/s, the resolution was 6.2 mm/s. This high flow resolution mainly occurred due to periodic flow disturbances generated in the wind tunnel (the standard deviation of the sensor signals was *σ*_*2*_ = 7.2 mV). If the ideal smooth flow was maintained under the mechanical noise generated by the airflow system, the sensor noise was the same as that measured under zero-flow conditions. In summary, the inherent resolution of flow velocity Sensor II was not less than 5.4 mm/s.

Sensor repeatability refers to the degree of inconsistency in the characteristic curve obtained from multiple full-scale tests of the same signal input to the sensor. Five repeated measurements were conducted on the output characteristics of the designed sensor, and the results are shown in Fig. 8f. This figure showed the distributions of repeated errors for the same sensor sample at different flow velocities. According to the bar graph, the overall repeatability error distribution of the sensor flow velocity was between 1.27% FS and 2.53% FS. According to further calculations, the average repeatability measurement errors corresponding to the thermal temperature difference and thermal loss in the sensor were 2.00% FS and 1.75% FS, respectively.

Sensor stability refers to the ability of a sensor to maintain its output characteristics after a period of operation. Tracking tests on the stability of the calibrated flow velocity sensors under laboratory conditions (24 °C) were conducted, and the test results are shown in Fig. 8g. The figure features the measured output results of the sensor under constant flow velocities of 0.83 and 3.5 m/s for 5 days. After calculation, it was found that the average stability errors of the flow velocity during sensor thermal loss and thermal temperature difference measurements were 4.93%FS and 3.71%FS, respectively. According to the graph, the stability error of the thermal temperature difference measurement of the sensor is smaller than that of the thermal loss measurement because the former was an indirect measurement that was less affected by external environmental factors. Compared with previously reported thermal flow velocity sensors (Table 2), the as-fabricated sensors in this article not only exhibited flexibility but also better or comparable measurement ranges and sensitivities. These advantages were indicative of the application potential of the proposed thermal flow velocity sensors.

**Table 2:** Comparison between different thermal flow velocity sensors

References	Flexibility	Flow range/(m/s)	Sensitivity/(mV/(m·s^-1^))
Cubukcu A. S.^13^	NO	0–5	N/A
Balakrishnan V.^14^	NO	0.2–9	91
Shen G. P.^15^	NO	<8	N/A
Cho M. O.^16^	YES	5–40	36
Xu W.^33^	NO	0–20	154.4
Xu W.^34^	NO	−26–26	58.2
Yi Z.^35^	YES	0–20	N/A
Steiner H.^36^	YES	0.5–3.5	N/A
Wei X.^37^	NO	0–2.5	642
Yunping L.^38^	NO	2–37	28.1
**This work**	**YES**	**0–30**	**467.3**

## Conclusion

In this work, a flexible MEMS thermal flow velocity sensor was developed with improved sensitivity and measurement range. The device sensitivity was improved mainly through three design parameters: (1) optimization of the distance between the heating resistor and the temperature measurement resistor; (2) design of a cavity structure at the bottom of the substrate to significantly reduce thermal loss caused by thermal conduction; and (3) use of a low-thermal-conductivity PI/SiO_2_ composite porous film substrate to reduce the heat transfer velocity between the heating resistor and the contact surface, ultimately reducing thermal loss. As a result, the measurement range of the sensor was expanded to 30 m/s with a resolution of less than 5.4 mm/s, and the average sensitivity was 467.31 mV/(m s^−1^). Compared to devices without a cavity structure made with a pure PI substrate, the average sensitivity was improved by 39%. This significant increase in sensitivity greatly enhanced the accuracies of wide-range velocity measurements. Therefore, the proposed flexible flow velocity sensor would have great significance in various fields, such as in aerospace, defense, and other fields that require a wide-range and high-sensitivity sensor that is suitable for complex surfaces.

### Supplementary information

Revised Supplementary Materials
