# Supplementary material for: Fabrication and characterization of high-sensitivity, wide-range, and flexible MEMS thermal flow velocity sensors
Source: Microsyst Nanoeng. 2024 Jul 22;10:102. doi: 10.1038/s41378-024-00740-2 (PMC11263615; doi:10.1038/s41378-024-00740-2)
Supplement: Supplementary file 1 — Revised Supplementary Materials [file 41378_2024_740_MOESM1_ESM.docx]

**Fabrication and Characterization of High-Sensitivity and Wide-Range Flexible MEMS Thermal Flow Velocity Sensors**

**Min Li^a, b, d, 1^Guangzhao Qin^e, 1^, Chen Jia^a, b, d, *^, Danyu Zhang^a, c, *^, Zhikang Li^a, b, d^, Xiangguang Han^a, b, d^, Shusheng Xu^d^, Libo Zhao^a, b, d^, Guoxi Luo^a, b, d,^ *, Cunlang Liu^a, c^, Ping Yang^a, c, d^, Qijing Lin^a, b, d^**

*^a^* *State Key Laboratory for Manufacturing Systems Engineering, International Joint Laboratory for Micro/Nano Manufacturing and Measurement Technologies, Xi’an Jiaotong University (Yantai) Research Institute for Intelligent Sensing Technology and System, Xi’an Jiaotong University, Xi’an 710049, China*

*^b^ School of Instrument Science and Techonology, Xi’an Jiaotong University, Xi’an, 710049, China*

*^c^ Xi'an Jingwei Sensing Technology Co., Ltd., Xi’an, 712000, China*

*^d^ Shandong Laboratory of Advanced Materials and Green Manufacturing at Yantai, Yantai, 264000, China*

*^e^ Brightstone Innovation (Yantai) Research Institute for Micronano Sensing Technology, Yantai 264006, China*

*To whom correspondence should be addressed: Chen Jia (jiachen0224@stu.xjtu.edu.cn), Danyu Zhang (dyzhang1213@163.com), Guoxi Luo (luoguoxi@mail.xjtu.edu.cn).

^1^Equal contributors.

**EXPERIMENTAL SECTION**

**Characterization**

**Thermal Stability Testing.** The STA 2500 thermogravimetric analyzer manufactured by Netzsch Instruments GmbH was used to conduct the testing in a temperature range of 30 to 800 ℃.

**Thermal Conductivity Testing.** The thermal conductivity testing of PI/SiO_2_ composite porous film was conducted according to the ASTM D5470 standard by using the Swedish Hot Disk TPS2500S. The sample size was a square of 40×40 mm, and each sample was tested three times.

**FTIR and XRD analysis.** The FTIR characterization was performed using the Nicolet iS10 infrared spectrometer from ThermoScientific (USA). Prior to testing, the samples were cut into 40 mm × 40 mm. The testing range for FT-IR was set from 600 to 2000 cm^-1^. XRD analysis was conducted using the D8 ADVANCE X-ray diffractometer from Bruker (Germany). The scanning angle range was set from 2θ = 10° to 50°, with a scanning velocity of 2 °/min. Before testing, calcine the sample to 850 ℃ and grind the residue into a powdered solid.

**Morphology analysis.** SEM analysis was performed to have insight into the morphology of the composite films by using HITACHI (Japan) SU8010 field emission electron microscopy. EDS analysis was performed using JEOL (Japan) TEM JEM-2100Plus transmission electron microscope. Before testing, the sample is ground to a powder like solid, dried, and ultrasonically dispersed in anhydrous ethanol.

**Measurement of Temperature Coefficient (TCR) of Thermistors**

Build a resistance temperature coefficient testing platform, including sensor prototypes, hot plates, desktop multimeters, and thermometers. Set the temperature of the hot plate to 30 ℃-180 ℃, measure the resistance of the thermistor every 10 ℃, and draw the temperature resistance curve as shown in **Figure S1**. As the temperature continues to rise, the resistance of the thermistor increases linearly. The temperature coefficients of each thermistor were calculated as follows: heating resistor: 0.1583 %/℃, temperature measurement resistor: 0.1609 %/℃, which basically conforms to the variation law of Pt resistance with temperature.


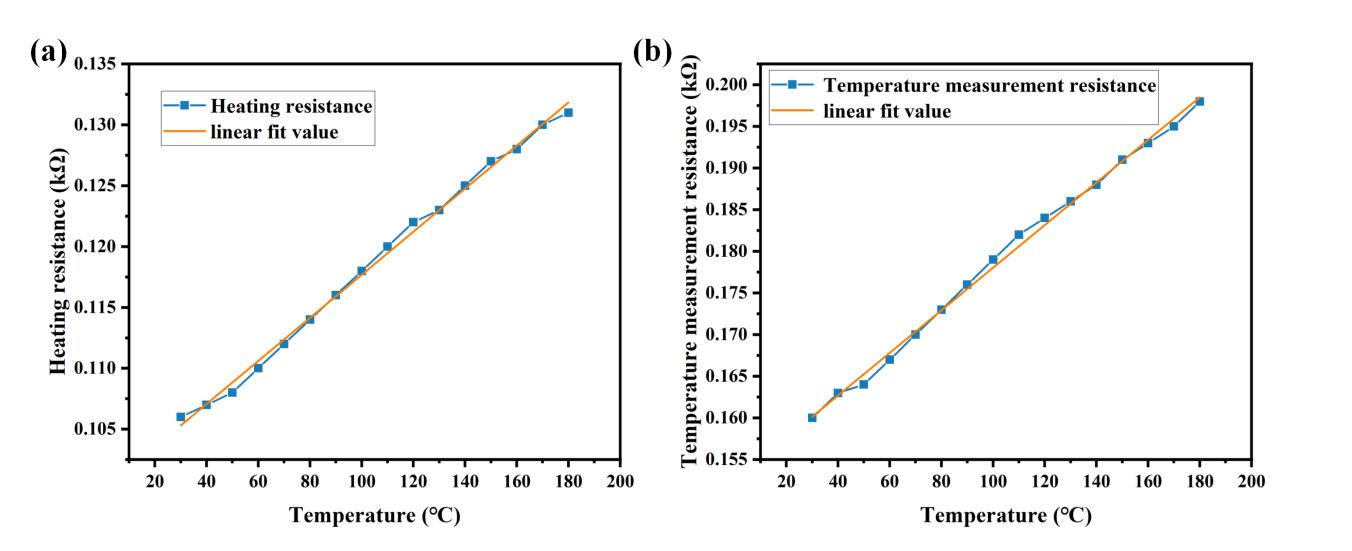


**Fig. S1** Measurement results of temperature coefficient of thermistor. (a) Temperature coefficient of heating resistor. (b) Temperature coefficient of temperature measuring resistor.

**SIMULATION**

In order to achieve simulation analysis of thermal flow velocity sensors, a simulation model is established as shown in **Figure S2** (a). Due to the temperature measurement resistor is mainly used for low flow velocity measurements, the flow velocity range is set to 0.2-3.2 m/s with a step distance of 0.2 m/s. The relationship between the temperature difference and flow velocity of the temperature measurement resistance pair at different distances was shown in **Figure S2** (b). And the sensitivity of temperature measurement resistance pairs at different distances was depicted shown in **Figure S2** (c).

By analyzing the relationship between temperature difference and flow velocity with different distances of temperature measurement resistors, it can be concluded that within a certain range, as the distance between the temperature measurement resistor and the thermal resistance increases, the velocity measurement range of the temperature measurement resistor decreases, but the sensitivity will increase. Taking into account the velocity measurement range and sensitivity of the temperature measurement resistance pair, it is necessary to reasonably set the relative position of the temperature measurement resistance pair and the thermal resistance. Therefore, two groups of temperature measuring resistors pairs were designed, and the distance between the two groups of temperature measuring resistors pairs and heating resistor is 300 μm and 500 μm respectively. In terms of velocity measurement scheme selection, when the flow velocity exceeds 2 m/s, the heat loss principle is used for velocity measurement. When the flow velocity is below 2 m/s, the thermal temperature difference principle is used for velocity measurement. The inner temperature measurement resistor pair is mainly used for velocity measurement. If extremely low velocity measurement is carried out, the outer temperature measurement resistor pair can be used.


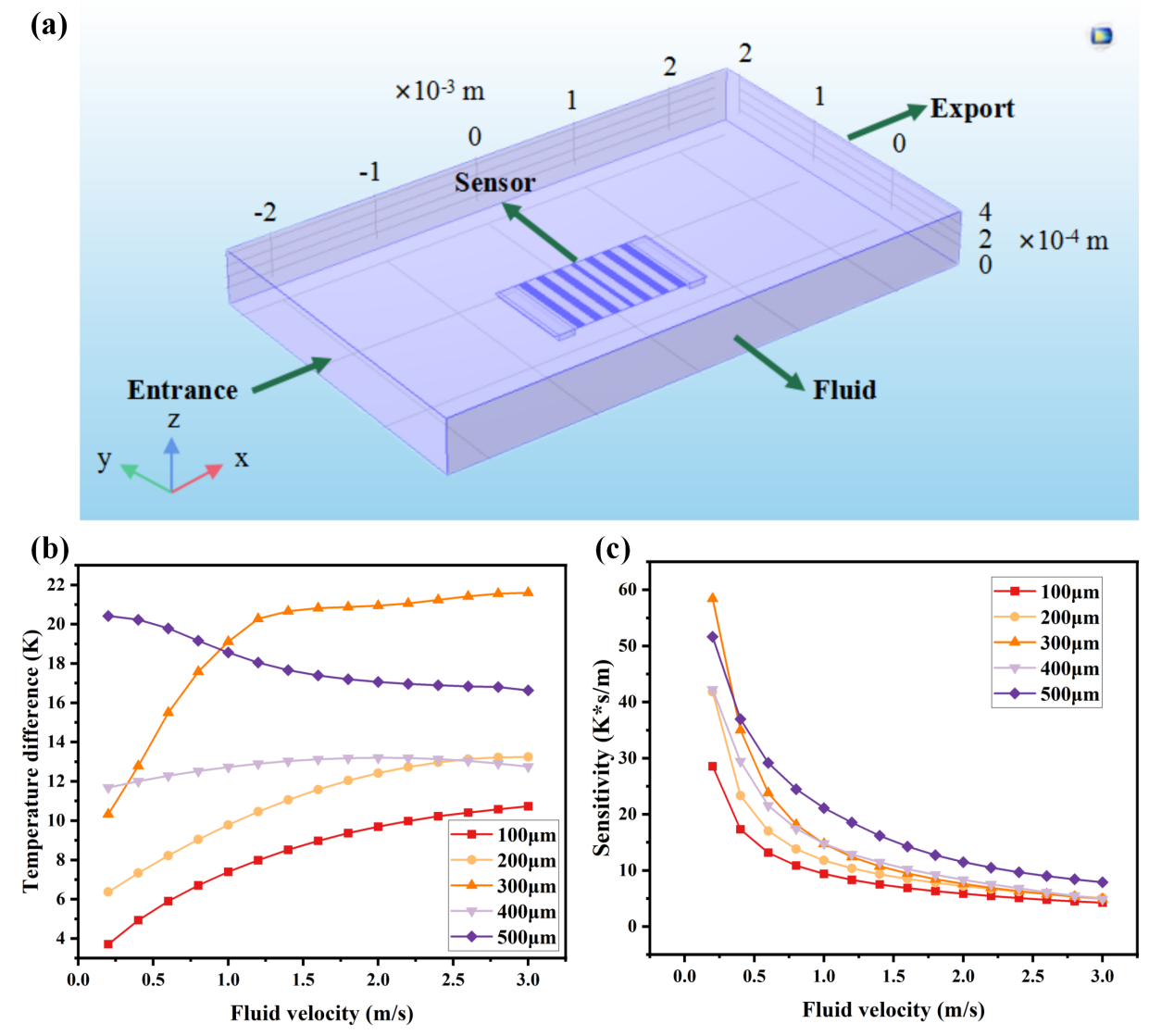


**Fig. S2** (a) Simulation model of thermal flow sensor. (b) The relationship between the temperature difference of temperature measuring resistors pair at different positions and flow velocity. (c) Relationship between temperature measuring resistors pair sensitivity and flow velocity at different positions.

**Multi-Channel Digital Output Circuit System**

The multi-channel digital output circuit system of the sensor mainly consists of ADC chip and microcontroller power supply circuit, three sampling circuit, CH340 chip for UART-to-USB conversion, a temperature compensation circuit, STM32 microcontroller, and STM32 upper computer software based on C^#^ development environment, as shown in **Figures S3** to **S8**.

Due to unstable power supply voltage, changes in component parameters, and changes in ambient temperature, temperature drift of the sensor can occur. The main reason for this is the change in temperature conditions. Due to the influence of ambient temperature on the resistance value of the thermistor on the arm of the Wheatstone bridge, the output of the bridge is not equal to zero, resulting in bridge imbalance. This study focuses on temperature changes and further optimizes the circuit from the perspective of sensor hardware compensation, using a single bridge temperature compensation method for compensation.

The circuit schematic is shown in **Figure S9**, where *R_a_* is the heating resistor, *R_b_* is the compensation resistor, *R_9_* is the variable resistor, *R_10_* and *R_11_* are constant resistors, and *R_a_* and *R_b_* are positive temperature coefficient thermistors with the same characteristics. When the resistance of *R_11_* is much greater than *R_10_*, most of the current will flow through *R_10_*, and the current flowing through *R_b_* will become very small, and the heat added by *R_b_* will also decrease. When the Wheatstone bridge in the figure reaches equilibrium, that is, the output voltage of the sensor reaches stability, it satisfies the following relationship:

$\frac{R_{9}+R_{b}}{R_{11}}=\frac{R_{a}}{R_{10}}$ （1）

Therefore, in this study, a thermistor with a high resistance value on the sensor was used as an environmental compensation resistor and connected to one bridge arm of the Wheatstone bridge. A high resistance thermistor was selected to minimize the current flowing through the resistor, thereby reducing the impact of environmental temperature and minimizing the drift of the circuit output voltage caused by external temperature changes.

The multi-channel digital output circuit system of the sensor is shown in **Figure S10**. During the operation of the sensor, the high-velocity output system and the low-velocity output system are subjected to three ADC sampling, filtering, amplification, and analog-to-digital conversion through the STM32 microcontroller. The three sampling circuits are composed of one high-velocity output system and two low-velocity output systems. The voltage of the high-velocity output system is represented by *V_1_*, with a measurement range of 2-30 m/s, and the signal is generated by the heating resistor. The measurement range of two low-velocity output systems is divided into extremely low velocity (<0.9 m/s) and low velocity (0.9-2 m/s), represented by *V_2_* and *V_3_*, and the signal is generated by the outer temperature measurement resistor pair (*R_1_*, *R_4_*) and the inner temperature measurement resistor pair (*R_2_*, *R_3_*), respectively. Finally, it is connected to the multi-channel voltage upper computer software through a data cable, allowing for real-time observation of the output voltage waveform and data storage.


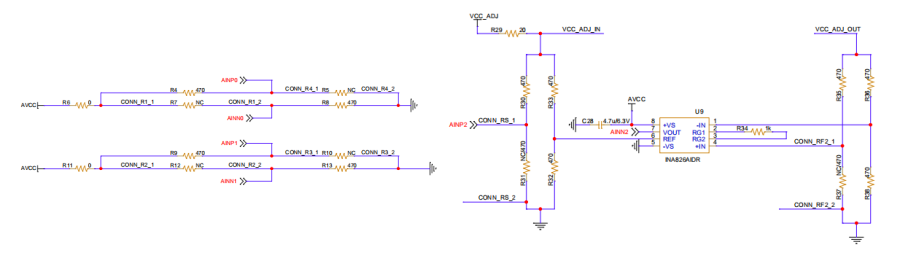


**Fig. S3** Design diagram of three-way sampling circuit for sensors.


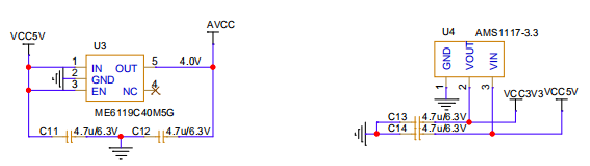


**Fig. S4** Design diagram of power supply circuit for sensors. (a) ADC chip power supply circuit. (b) Microcontroller power supply circuit.


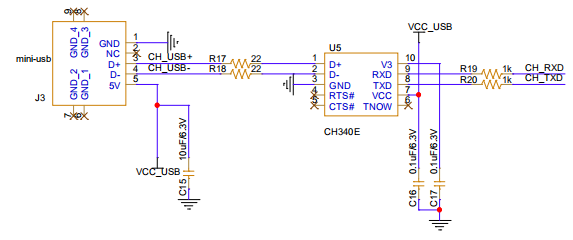


**Fig. S5** Design diagram of UART-to-USB chip for sensors.


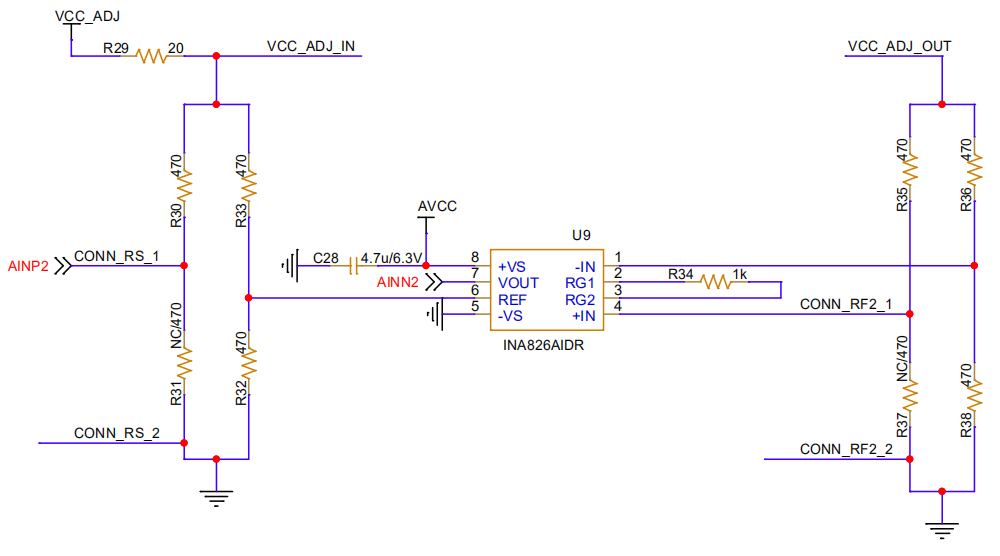


**Fig. S6** Design diagram of temperature compensation circuit for sensors.


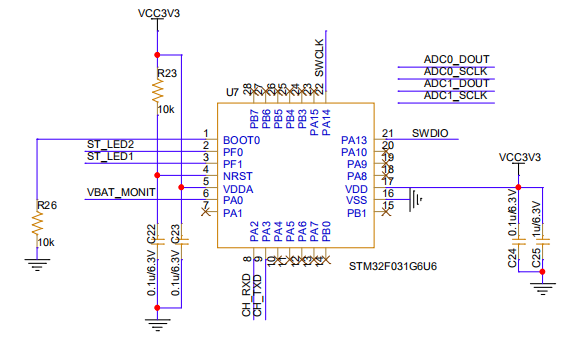


**Fig. S7** Design diagram of STM32 microcontroller for sensors.


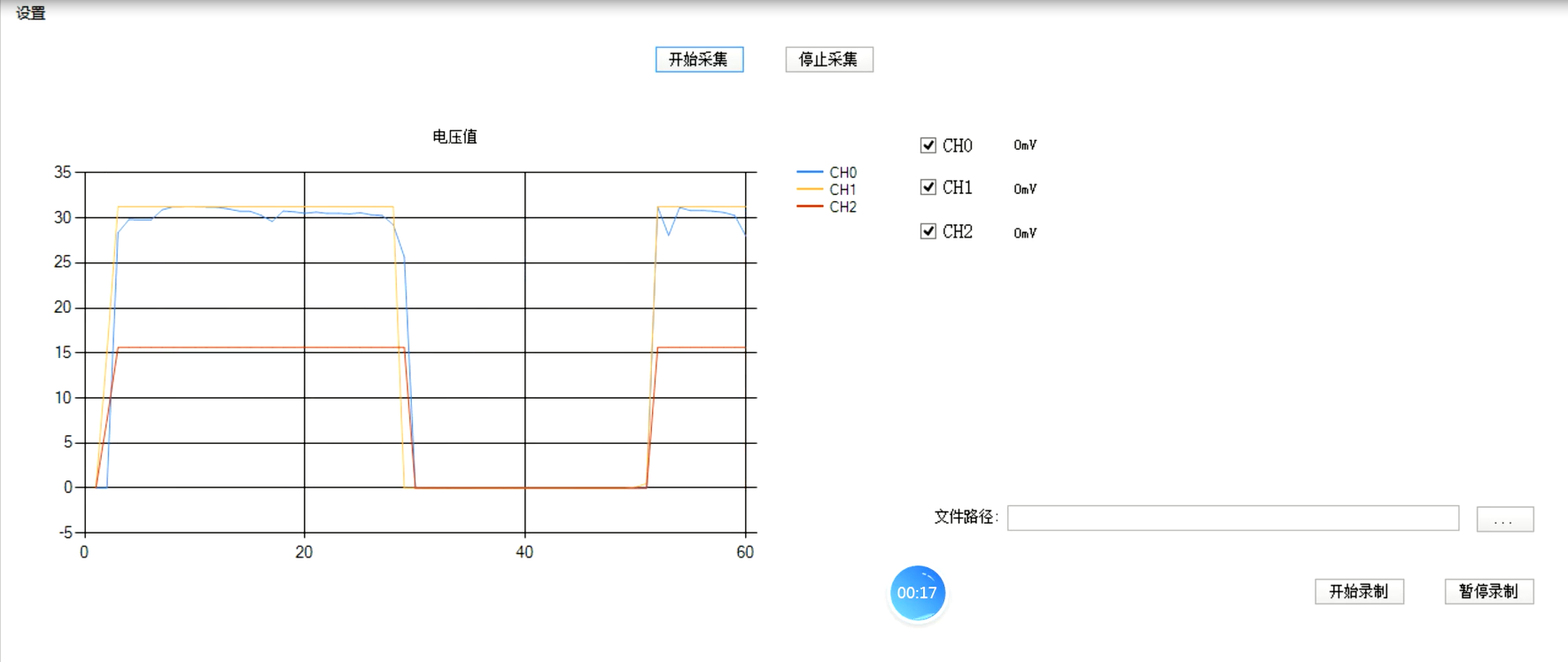


**Fig. S8** Design diagram of STM32 upper computer software for sensors.


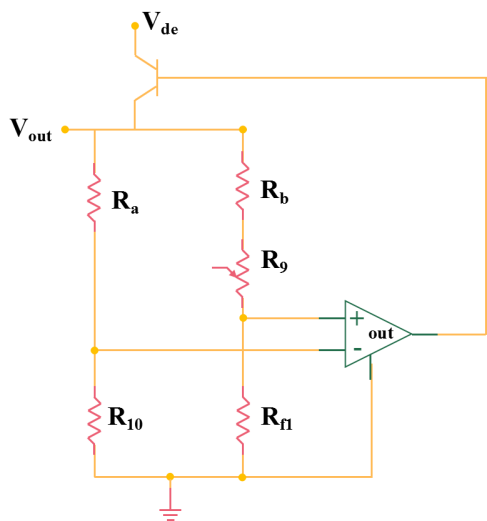


**Fig. S9** Schematic diagram temperature compensation circuit for sensors.


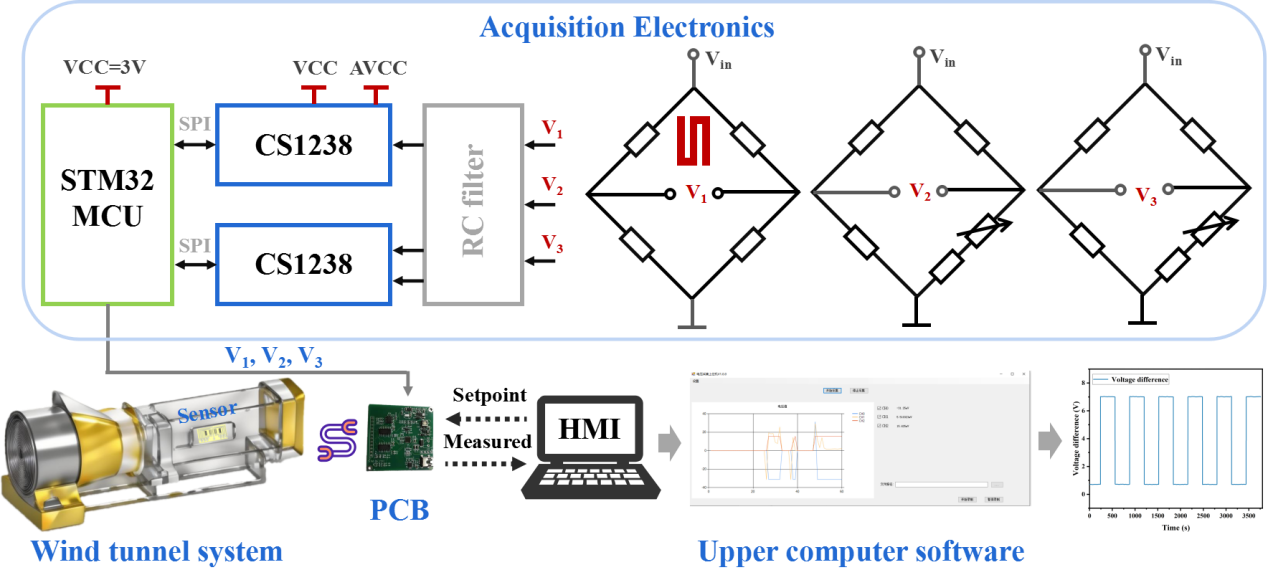


**Fig. S10** A multi-channel digital output circuit system for sensors.
